# Approaches and tools to measure individual-level research experience, activities, and outcomes: A narrative review

**DOI:** 10.1017/cts.2025.10076

**Published:** 2025-08-11

**Authors:** Brenda M. Joly, Carolyn Gray, Julia Rand, Katy Bizier, Karen Pearson

**Affiliations:** 1 Public Health Program, Muskie School of Public Service, University of Southern Maine, Portland, USA; 2 Cutler Institute for Health and Social Policy, Muskie School of Public Service, University of Southern Maine, Portland, USA

**Keywords:** Researcher investment, researcher experiences, research career, measurement, evaluation metrics

## Abstract

Strengthening the research workforce is essential for meeting the evolving needs and challenges in the health and biomedical fields. To do so effectively, it requires an understanding of how the experiences of a researcher shift over time and how one’s research career evolves, particularly as supports are put in place to foster research. This narrative review provides a summary of published individual-level assessment measures and survey tools from 2000–2024. All measures were abstracted, classified, and coded during analyses to describe the areas of focus, and they were organized into one of six research categories. The review identified a range of measures and methods across all categories. However, the measures were often narrow, focused on outputs, and not ideal for assessing the full range of experiences a researcher may have throughout their career. The most common metrics were related to research productivity and bibliometric measures. Our review of survey tools revealed a gap in comprehensive approaches available to assess an individual’s research experience, efforts, supports, and impact. As efforts expand to evaluate and study the research workforce, tools that focus on a broad range of individual-level measures, tied to specific underlying constructs and drawn from the literature, may prove useful.

## Introduction

The need to provide research support to early career scientists and clinicians is well documented in the literature [1–9]. In the United States, the demand for skilled researchers in the health and biomedical field is expected to increase as the gap between supply and demand continues to widen [10–12]. Studies suggest that the path to research independence takes longer now than in previous decades, resulting in what is known as a “holding zone [6].” Efforts have been underway to promote diversity in the workforce [13] and to attract and build a pipeline of researchers to address contemporary health issues and challenges [12]. Several federal agencies, including the Veterans Affairs, the National Institutes of Health (NIH), and the Agency for Healthcare Research and Quality, provide career support, training, and mentoring [4]. One example is the support NIH provides for clinical and translational researchers through two national programs. These programs, known as Clinical and Translational Science Awards (CTSAs) and Institutional Development Award Networks for Clinical and Translational Research (IDeA-CTR) seek to improve health by fostering new research and accelerating its use. A key strategy of these programs relies on building, training, and strengthening the research workforce and ongoing efforts to evaluate the success of these initiatives based on published guidelines [14].

While efforts to measure individual-level changes in the health research workforce remain a priority, there are few tools and systematic approaches to do so. Bilardi and colleagues [15] found a lack of comprehensive tools that are widely applicable and able to provide standardized and consistent measures. This narrative review describes the characteristics and focus areas of individual-level metrics designed to assess research experience, activities, and outcomes. It uses an organizational framework to categorize the measures into one of six areas. The review also summarizes the use of common bibliometric indicators and survey tools, identified and flagged during the review process, to provide further details on their focus areas and use.

## Methods

### Review and abstraction process

An initial review was conducted in 2021 to scan the published literature for tools and frameworks associated with return on investment (ROI) particularly related to federally funded programs such as the CTSAs. Results indicated relatively few studies that report implementing an evaluative framework to capture the individual-level productivity and impact of research across the career path. That search provided the foundation for the current study reported here, whose purpose was to identify any individual-level assessment tools and measures and determine gaps and opportunities for development of future measures.

**Figure 1.**
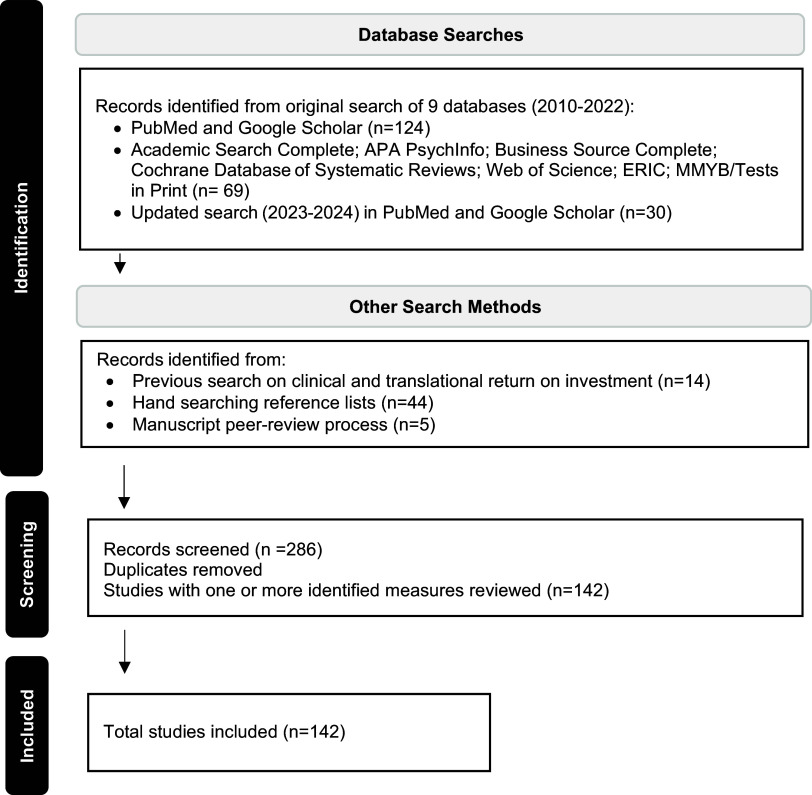
Search Process and Results.

A literature search was conducted in 2022 by an experienced librarian to identify publications that included indicators or measures related to an individual’s research-related experience and activities, as well as the outcomes or impact of their research. A second professional librarian provided input in December 2022 on search strategies and additional databases in the disciplines of psychology, education, and engineering, to identify articles, including review articles that focused on assessing or measuring research productivity and/or research capacity, especially for junior faculty on a research career trajectory. The aim was to identify as many tools and individual metrics as possible that were relevant to clinical and translational research in order to create a new, more comprehensive tool. The following questions framed the search: what are the key factors that enable successful research productivity and how is that measured in this discipline? Are there specific assessment tools (e.g., surveys) pertinent to each discipline? The databases searched included: PubMed, Academic Search Complete, Business Source Complete, Cochrane Database of Systematic Reviews, ERIC, Ei Compendex, MMYB with Tests in Print, APAPsychInfo, Web of Science, and Google Scholar. All databases were searched for publications from 2010 through 2022. Search terms included combinations of the following key terms: research capacity, research investment, researcher productivity, translational research, research assessment, assessment tool, and the wild card term research method*. As seen in Figure 1, 124 publications were identified through an initial search of PubMed and Google Scholar. The database search of non-health disciplines yielded an additional 69 articles. A total of 44 publications were pulled from the reference lists of relevant articles scanned by the lead author. Fourteen articles retrieved from a previous internal study (unpublished) focusing on clinical and translational research ROI were included in this review. All publications were exported into Endnote and an annotated subject bibliography was produced for team review. Based on our analysis and coding approach (see below), we ran our search again in 2023-2024 in PubMed and Google Scholar, yielding an additional 30 articles to fill in gaps identified in the preliminary search. A final peer-review yielded five additional articles for inclusion.

The publications were reviewed and abstracted using a standardized approach (described below) and three separate spreadsheets. The first spreadsheet recorded all individual-level measures (e.g., serving as a peer reviewer). The second spreadsheet recorded all bibliometric indicators identified during the review. The third spreadsheet captured survey tools that were also identified and flagged during the review. All survey tools (named or unnamed) that included a structured questionnaire with a set of items (scale) or set of scales measuring constructs were abstracted. The tools were reviewed, regardless of whether they reported psychometric testing. However, any tools that were strictly qualitative, inadequately described, or or omitted the item wording and response options were excluded, as were post-program surveys, evaluations, or tracking systems not designed to measure underlying constructs.

### Individual-level measures

**Figure 2.**
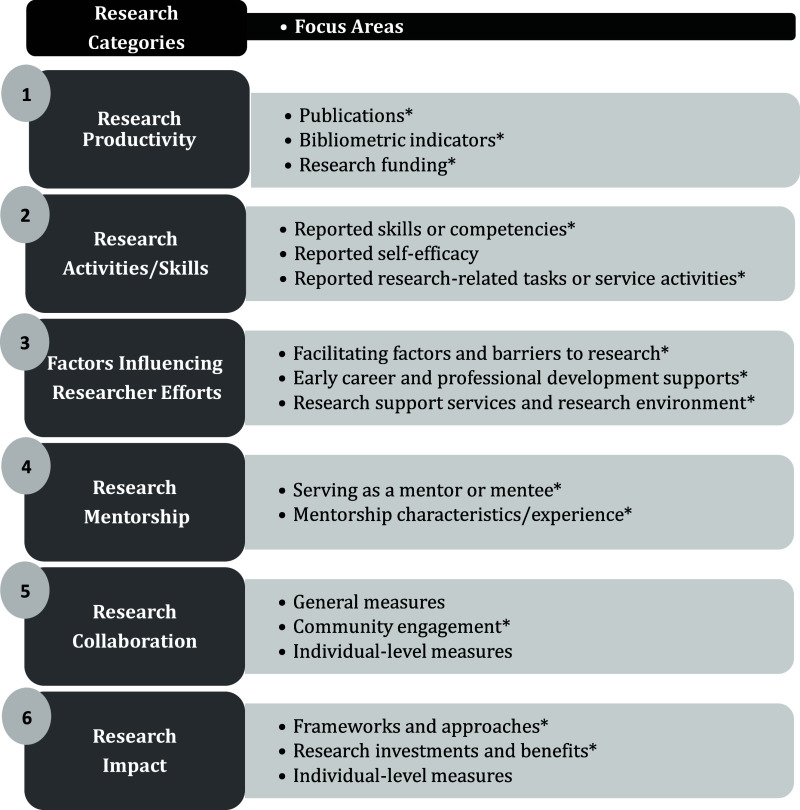
Assigned Research Categories and Focus Areas Used in Coding. (Note: * Codes developed *a priori*).

Figure 2 depicts 17 focus areas identified by the lead author from the literature for coding purposes. The codes were classified into six overarching research categories. All but four codes were determined *a priori* based on areas deemed relevant to the evaluation of NIH-funded clinical and translational research initiatives. Each measure was recorded under one of the 17 focus areas. The individual-level measures, citation, and the question wording and response options (when available, and as applicable) were catalogued in the spreadsheet..

### Bibliometric measures and survey tools

The data abstraction for all flagged bibliometric indicators included the measure name, definition, focus, and type (e.g., individual or organizational), as well as the date published, and the author and complete citation. The following information was abstracted for each survey tool: the year of publication, the authors and citation, the study location, the name of the tool (if applicable), the total number of items, the item wording and response options, the respondents, a description, and any reported validation efforts.

## Results

### Individual-level measures by research category

The results are discussed within the framework of the six research categories depicted in Figure 2.

### Research category #1: research productivity

The most frequently used measures focused on the concept of research productivity and they were typically assessed by exploring publications and research grants or funding. As noted below, most approaches relied on quantifying publication efforts (e.g., total publications), and external grants (e.g., total number of awards). However, in a few instances, algorithms were used to measure the productivity of researchers. For example, propensity score matching was used to compare funded and non-funded researchers with impact scores [16]. Additionally, Wootton and colleagues [17] developed a research output score based on the sum of three measures: 1) peer reviewed publications, 2) research grant income, and 3) PhD student supervision. In this case, peer reviewed papers were assigned publication “points” based on publication year, journal impact factor (JIF), and author position. Grants were included if they were awarded competitively and within a given timeframe and they were weighted by role and took into account the grant “income.” Points were given for supervising PhD students when a thesis was aligned with their research and occurred during the year in review [17]. Finally, one study explored faculty research productivity by calculating the number of publications during the first few years of an academic appointment, prior to an initial promotion, and following promotion. This study also explored research productivity to determine associations based on rank and length of time at a given rank [18].

#### Publications

Overall, the most frequently published productivity measures were related to self-reported publications [1,3,17–23] and the use of bibliometric indicators based on existing databases (described below) [16,24–45]. Publications were measured by computing the total number overall and by type (e.g., peer-reviewed) [4,21,26,29,34–36,46], tallying articles published during a set time period [20,21,27,45], focusing on articles linked to funded projects or training programs [5,21,47], analyzing the publication date and sometimes comparing it to when specific funds were received [20], assessing the impact factor of top tier journals, and weighing authorship order and authorship collaboration [4,28,31,34,41,48], Self-reported publication measures were typically compiled through a review of curriculum vitas or survey questions [3,18,23] or via a publication list [21].

The JIF is another common approach that has been used to explore publications. Journals with high impact numbers have been used as a vehicle to secure grants, tenure, and raise awareness of translational research [49]. Using the JIF along with the h-index (described below) is useful in assessing an individual researcher’s impact across their career trajectory. Of note is the current movement away from the JIF, instead promoting a more comprehensive assessment of research quality that goes beyond the basic reliance on the influence of the journal in which the studies are published and looks at the merits of the individual studies. To that end, the San Francisco Declaration on Research Assessment provides a set of recommendations for researchers, institutions, and publishers in the measurement and dissemination of research publications [50].

#### Bibliometric measures

In the last two decades, bibliometric indicators have been used to assess metrics focused on lifetime publications, citations, authorship order, publication rates, and scholarship quality [17,30,34,36], A number of indices have been created to compute scores or values based on publication and citation data. In 2005, the *h*-index was published as a new approach to characterize research output that could be used across scientific disciplines [32,51]. This index is among the most commonly reported and it relies on both the publication and citation record of an individual [52]. As seen in Table 1, several additional approaches using bibliometric data now exist including the: *g*-index [24], integrated researcher productivity index [31], relative citation ratio [53], scientific quality index (SQI) [40], scholarly productivity index [44], category normalize citation impact (CNCI) [43], Ab-index [54], fractional scientific strength index [24,55], and the future *h*-index [25]. These measures largely focus on productivity by exploring an individual’s cumulative publication and citation record using different assumptions and algorithms.

**Table 1.** Bibliometric measure

Year	Authors(s)	Name of Measure	Type of Measure	Definition or Focus
1997	Huettner *et al*. [33]	Research Productivity Measures of an Academic Department	Organizational-level	An economic model of research productivity based on the supply and demand of journals, with the following criteria: 1) annual journal publications per faculty, 2) acceptance rates in top five journals, 3) number of authors per article, 4) equivalent pages per article and per author/per year, 5) average size of NSF grant awards, 6) number of subscribers to top 5 discipline-based journals.
2005	Hirsch [32]	*h*-index	Individual-level	The *h*-index is an indicator of the impact of a researcher on the development of his or her scientific field and is defined as the maximum number *h* of works by a scientist that have at least *h* citations each. For example, if your *h*-index is 10, then you have 10 publications with at least 10 citations each.
2008	Duffy *et al*. [31]	Integrated Research Productivity Index (IRPI); Author-weighted scores	Individual-level	The IRPI measures individual productivity by statistically combining an individual’s author-weighted publications multiplied by the mean number times cited by other publications, divided by years since first publication (Y) into a comprehensive score. Author-weighted scores are computed by assigning points to an individual on the basis of how many authors are on a particular publication and the author’s place in the order of authors, with each article allotted a total of 1 point.
2013	Abramo *et al*. [24]	*g*-index	Individual-level	A variant of the *h*-index, the g-index measures the global citation performance and research productivity of an author, and is defined as the highest number *g* of articles that together receive g^2^ or more citations.
2013	Biswal *et al*. [54]	Absolute Index (Ab-index); Productivity Index (Pr-index)	Individual (Ab-index) Organizational level (Pr-index)	The Ab-index reflects an individual scientist’s contribution to the field and calculates the author’s partial contribution to all papers authored or co-authored. The first author and the corresponding author get equal credit while the rest of the co-authors get credit in a decreasing arithmetic progression. To compare individuals of different age groups, the Pr-index is calculated as the ratio of the Ab-index and the number of scientific years spent to generate the publications. The Pr-index of an institute will be the sum of the Ab-indices earned by individual researchers of the institute per year.
2013	Tsai *et al*. [47]	Research Resident Training Program	Individual & Organizational-level	Primary outcome in determining the success of the RRTP was whether each resident entered a postdoctoral research fellowship after graduation and percent of RRTP residents publishing in a peer-reviewed article during or within 2 years of graduating.
2015	Noble *et al*. [37]	Scholarly Productivity	Individual & Organizational- level	Defined as the number of publications, number of citations, and *h*-index of individual academic staff members; aggregated by academic institution for organizational productivity measure.
2016	Hutchins *et al*. [53]	Relative citation ratio (RCR)	Individual-level	Article-level calculation to show the field- and time-normalized citation rate, benchmarked to 1.0 for a typical (median) NIH paper in the corresponding year of publication. Fields are defined for each article by using its co-citation network.
2016	Walters G. [44]	Scholarly Productivity Index	Individual & Organizational-Level	Based on 6 independent variables: 1) total number of publications, 2) total number of first author publications, 3) citing articles to total publications with all self-citing articles removed, 4) total number of citations to all first author publications, 5) total publications h-index, 6) first author h-index
2017	Pluskiewicz *et al*. [40]	Scientific Quality Index (SQI)	Individual-level	Calculated by the addition of the percent of papers cited ≥ 10 times and the mean citation score (excluding self-citations and the citations of all co-authors)
2020	Szomszor *et al*. [43]	Category Normalized Citation Impact (CNCI)	Individual & Organizational-level	Calculated by dividing the actual count of citing items by the expected citation rate for documents with the same document type, year of publication and subject area.
2021	Bautista-Puig *et al*. [28]	Scientific Output and Productivity; Visibility	Organizational-level	To quantify the impact of researchers’ financial bonuses on scientific output over time. Calculated by the total number of publications and the cumulative average growth rate. Productivity is defined as the ratio between number of researchers and number of documents. Visibility is the number of publications listed in Journal Citation Reports in first quartile journals pre- and post-bonus.
2022	Paul *et al*. [39]	Research Productivity by Discipline	Individual & Organizational-level	To measure research productivity of marketing scholars and marketing departments. Two metrics are used: total number of publications across 4 top-tier journals in the field and a fractional publication score which includes a ranked author order.
2022	Lai *et al*. [34]	Research Performance: 1. Average SJRs (SCImago Journal Ranks), 2. Lifetime Citations, 3. Lifetime First Author Publications	Individual-level	To measure lifetime bibliometric research performance norms of academic psychologists in the UK. 1. Average SCImago Journal Ranks is defined as the average number of weighted citations received during a selected year per document published in that journal during the previous three years. 2. Lifetime citations is the total number of citations accumulated within a given time span. 3. Lifetime first author publications is the sub-set of lifetime Scopus publications for which the academic researcher was listed as the first author.

#### Research funding

There was a lack of consistency in the ways in which funding data were obtained ranging from surveys that included yes or no responses, Likert-type scales options, or open-ended questions. Some approaches relied on a review of existing documents, administrative records, or curriculum vitas. Overall, the number, amount (e.g., total research awards in dollars), type (e.g., competitive, extramural, foundation), and source of awards (e.g., federal, sponsor name) received were the most frequent measures used to assess research funding [1,3,5,20,27,29,36,56–61]. These measures represented the volume of funding (e.g., how many research grants, the total award amounts) [4,19,56,57], the first time a particular type of award was received [58], the consistency of funding over time [5,62], the nature of the award [5,16,21], and the prestige of the funding source [3,8,20,57,59]. Several studies also investigated the researcher’s role (e.g., Principal Investigator), proposal submissions, and award decisions including those receiving and not receiving funding [4,5,8,20,22,26,27,57,60,63,64]. A few studies explored potential factors impacting subsequent funding such as career development training [5,59,64,65]. More recently, studies have focused on the number and type of funding received among early-stage researchers [3,8,59,60,64]. For example, Chou and colleagues [3] surveyed new and early stage faculty who were the recipients of pilot funding through the Oklahoma IDeA Network of Biomedical Research Excellence Research Project Investigator award program.

### Research category #2: research activities and skills

This group of measures focused on a number of activities in which researchers are involved throughout their career and they were often aligned with the productivity measures noted above. For instance, the most common activities were related to *publishing* and *securing* funding through grant writing [19,29,66–68]. Additional activities were linked to presenting research, including early career experience delivering oral presentations [69], and submitting unsuccessful grant proposals and articles that have not been published [29]. The literature also identified items focused on participation in external NIH advisory groups or other committees [5] serving as a peer reviewer for papers or grants [29], serving in an editorial role [4], teaching [29,66], training or mentoring students or post graduates [8,63,70], participating in scientific or professional societies [5], submitting ethics and regulatory applications [42], receiving prizes or awards tied to research [7,24], and contributing to new guidelines [39].

Several research activity measures were also used to assess individual skills, or the self-efficacy of selected research skills related to: the conceptualization of a research project [62,64], proposal or grant writing [60,62], regulatory compliance [60,65], management and oversight [64–66], the collection, recording, and analyses of data [65], and the dissemination of findings [64], including report development and presentations [65].

### Research category #3: factors influencing researcher efforts

#### Facilitating factors

Several studies focused on motivators to do research [63,67,68] and the characteristics, beliefs, and roles of those involved in research, including individual attributes [63,69], research self-efficacy [63], administrative roles [70], those in top positions [71], and those who face greater challenges competing for research funding [2]. For example, Ommering and colleagues [69] studied the association between academic success and measures of motivation and research self-efficacy among first year medical students based on their grades in a research-related course. They included nine items measuring intrinsic and extrinsic motivation for research (e.g., doing research is interesting, fun, challenging and is useful for my resume), and three items related to self-efficacy (e.g., I feel I am competent enough to do research).

A range of factors linked to one’s research skills, quality, success, attrition, and overall productivity, volume, and scholarly impact were also explored [2,52,70,72,73]. For example, one study examined the role of age, academic rank, self-confidence, years of research experience, and teaching load [70]. Common facilitating factors promoting research included: compensation [51,72], start-up support [74], career satisfaction [72], percent of time dedicated to research—known as protected, uninterrupted, or quarantined time [57,68,74], prior research socialization and opportunities [15,51], laboratory experience [74], skills training [68], and early career research engagement [75]. Our review also revealed that work considered personally meaningful, with a flexible schedule and collaborative team environment promoted satisfaction. For instance, Kalet and associates [71] surveyed women who participated in the Clinical Scholars Program sponsored by the Robert Wood Johnson Foundation. They found that flexibility was critical in their decision to remain in an academic career given greater perceived freedom to care for family, attend school events or appointments. Dzirasa and colleagues [72] published a case study of a pilot training program for a single MD/PhD graduate to explore research engagement, unyoking clinical and research milestones, and a path to independence based on an integrated training model, dedicated research time, space, resources and salary support, as well as mentorship. The program proved successful resulting in NIH funding and research independence within 3.5 years versus the average nine-year schedule typical for MD/PhDs [72].

#### Research barriers

Impediments included uncompensated research costs as well as lack of resources or supports [51]. Increased competition for funding, administrative burdens, student debt, and issues related to regulatory compliance, work-life expectations, and reduced supports were also cited as obstacles to research for clinical scientists [73]. Goldstein and colleagues [73] reported a major impediment for surgeon-scientists is the long hours required resulting in difficulty achieving a work-life balance and their paper underscored the need for strong social support. One study of medical school faculty found that the strongest predictor of intent to leave academic medicine was problems balancing the demands of family and career [74]. The lack of timely and constructive support from departmental leadership was also linked to attrition [74].

#### Early career and professional development supports

A number of articles explored early career plans and development opportunities. For example, one study asked medical students how extensively they planned to be involved in research during their career [58]. Other studies focused on the characteristics of training programs including the duration [63], timing (e.g., during schooling), components (e.g., laboratory experience), and participants (e.g., research staff and Principal Investigators) [75]. A few studies focused on the quality and culture of graduate training [76], academic rank of participants [77], and personal factors linked to training participation [78]. More recently, several studies have begun to focus on individual groups of researchers who have participated in tailored career development programs or opportunities including: respiratory disease young investigators [22], Perinatal Research Society scholars [64], clinical investigator training programs [71,79], medical students [58], MD/PhD programs [80], and new or early stage faculty or scientists [3,5,8,81–84].

#### Research supports and environment

The literature in this area is largely descriptive, lacking specificity in terms of how items are measured and collected. A number of research supports were identified including assistance provided by grant programs and staff [1], leadership support [85], financial and social supports [73], organizational supports [86], and structures that facilitate interdisciplinary work and encourage work with external partners [85]. Additional institutional practices that define clear roles, have fair decision-making, adopt agreed upon processes for reaching agreement, and offer rewards were also identified [22,77,85,87–90]. Added supports were characterized by focusing on the research systems in place at a given organization [87], the managerial actions related to resource allocation [73], the research culture of specific departments [69], and the overall research culture of the organization, including the mentoring climate [15,70,91,92]. For example, the literature on translational research revealed the importance of organizational cultures that support collaborative work and entrepreneurial science skills [88]. Fudge and colleagues [93] found a reported shift in attitudes among scientists who perceived an advantage to securing funding for translational research based on becoming more “entrepreneurial” or working in an entrepreneurial organization.

### Research category #4: research mentorship

The literature on research mentorship largely focuses on characteristics, components, value, or benefits of mentoring programs, as well as the effectiveness of research mentors. Common areas of research inquiry include the quality of the mentor/mentee relationship [94], mentee satisfaction and retention [95], mentorship hours received and the types of research projects in which mentees participated [92], as well as the mentoring skills of those providing research mentorship [94,96–98]. A few studies have underscored the importance mentoring programs have had on women, medical school faculty, and underrepresented groups [74,91,99] and the value of creating a career map or academic development plan [99,100]. One study conducted by Bice and associates [94] examined faculty productivity by calculating composite variables to assess graduate mentorship with a focus on hours spent per week with the advisor, the number of projects with the advisor, the amount of communications with the advisor (e.g., never, once a month), and the perceived supportiveness of the advisor (e.g., extremely to not supportive). The findings suggested the number of hours spent was less important than involvement in meaningful research projects where students can participate in multiple aspects of the research.

### Research category #5: research collaboration

Research collaboration is commonly measured by operationalizing various constructs based on participatory approaches and models. The measures tend to focus on research projects, including the processes and outcomes of the work [101]. The metrics also tend to explore the relationships, climate or expectations of partners [102], as well as the attributes [103], and many of the measures focus on the actual partnerships (e.g., community-academic) or the participating organizations (versus individuals). For example, Greenwald and colleagues [102] identified metrics that included self-reported measures to assess organizational activities, communication among partners, information exchange, resource allocation and values, and policy or advocacy efforts. Another study measured the stages of community engagement to track the progress of research projects over time [104]. Proposed metrics to assess community-engaged clinical and translational science research have been organized into process measures focusing on research activities, and outcome measures linked to the contributions of community-engaged research [105]. Common measures included the synergy between research and community interests, priorities or concerns, as well as the partnership dynamics and outcomes based on research projects [100–102,101,105–107]. Additionally, efforts to include the community in research [105,106,108], and the dissemination of findings were also cited in the literature [66,109,110]. In a review of community engagement measures conducted by Eder and colleagues [105], CTSA organizations reported collecting a number of process measures such as the number and type of community members engaged, the number of projects that receive support from a community engagement core, the number of projects that seek input from the community, and the number of community–academic interactions during a given project, to name a few.

Efforts to assess research partners or collaboration at the individual level are less commonly reported and the literature tends to focus on tracking engagement, enhancing partnerships, the benefits to researchers, and perceptions about the collaboration. Trochim and colleagues [111] created a Researcher Form to collected self-reported data based on four areas: 1) satisfaction with collaboration, 2) impact of collaboration, 3) trust and respect among partners, and 4) attitudes about transdisciplinary research. Other studies have explored the benefits of collaboration on scientists’ productivity and early career measures by assessing team composition [112–115]. Several measures to track collaborative efforts or team science often focused on publications [108–110] including indicators that assessed a researchers’ contributions and the number of distinct institutions with which researcher’s co-authors are affiliated [41]. Partner contributions to grantsmanship, project implementation as well as collaborative service, teaching, and leadership efforts were also reported [115]. Metrics for enhancing community-academic partnerships included measures related to role clarity, inter-professional research teams, and use of quality improvement and practical trials, as well as skill-building, partnership development, and outreach to public health agencies [113,116].

### Research category #6: research impact

Literature on the impact of health research investments are largely based on conceptual frameworks that reference individual-level, community-level, organizational-level, or societal-level results [117–122]. For decades, the importance of using data to improve decision-making around funding investments in research has been acknowledged. For example, a 1986 Technical Memorandum authored by the Office of Technology Assessment and commissioned by Congress, provided an assessment of the use of quantitative approaches to help guide research funding decisions [123]. The authors emphasized that despite the subjective natures of quantitative measures, they are invaluable for analytical comparisons and describing trends. In their narrative review on measuring research impact, Greenhalgh and colleagues [124] conclude that there is not one standard method for measuring research impact; different purposes require different approaches. One common approach for assessing clinical and translational research impacts is the Translational Science Benefits Model with over 50 publications in PubMed citing this work [122].

Our review of the literature found that, in general, the benefits or impacts of health research are often captured as efforts that: influenced clinical procedures, guidelines, or testing [122], led to career advancement [48], contributed new knowledge to the field [117,119,121], led to future research and development [119], resulted in new methodology, innovative discoveries, drugs, software, medical devices or diagnostics [118,122,125], led to economic benefits (e.g., reduced social costs of chronic disease) [122] and influenced policymakers, decision-makers, and leaders [117,118,121,122,126,127].

Indicators specifically linked to the benefits and impact of health research included: improved health services, clinical practice and patient outcomes [118,119,122,127], the development of health and social service guidelines, informed public health policies, the creation of new health education material, enhanced advocacy [118,122], decreased medical errors and adverse drug effects, improved adoption of adherence to new guidelines and best practices, improved health of patient panels, strengthened patient-clinic relationships, improved continuity of care and overall safety, improved service delivery, and improved distribution and equity in health care access and quality [118,120,122]. Economic benefits and public health outcomes were also identified including enhanced quality of life and an increase in life expectancy [118,120,122].

Finally, ROI was a common theme used to describe the impact of research [118,128]. A review of several impact frameworks and approaches has been reported elsewhere including economic studies [129], and applications of various approaches used to assess the paybacks or returns tied to research investments have been well documented [121,128–133]. However, the literature on individual-level measures to assess research impact is limited. For instance, in 2009, the Canadian Academy of Health Sciences published a report describing a framework with a list of 54 indicators and corresponding metrics to assess ROI in health research, including aspirational measures [118]. In this report, they generated a list of five impact categories: 1) advancing knowledge, 2) capacity building, 3) informing decision-making, 4) health impacts, and 5) broad economic and social impacts. Each indicator was aligned with recommendations for application at the individual, group, institutional, regional, or national level. Of the 54 indicators, 21 were relevant at the individual level and only one “impact” indicator (self-reported continuity of care based on patient surveys) was appropriate at the individual level. The remaining indicators proposed to assess health impacts and broad economic and social impacts were applicable at the provider, organization, regional, or national level.

### Survey tools

There are a range of survey tools designed to assess one or more aspects of a researcher’s experience, activities, and outcomes, yet relatively few publications provide the item wording and response options used to operationalize the constructs. During our review, we identified and flagged 11 survey tools published from 2001 to 2022 with specific individual-level measures that met our criteria. As seen in Table 2, seven of the 11 tools were validated [68,87,112,136–139]. The surveys ranged in length, from seven to 150 items. In general, respondents were faculty, clinicians, health professionals or researchers. In one case, policymakers were surveyed. While the scope varied, most of the tools were narrowly focused on research skills, self-efficacy, or attributes of the researcher, and nearly all included one or more measures assessing research activities. Four of the tools were developed in the United States [20,87,112,138] and three of the four were administered to medical school faculty or clinical researchers [20,87,138]. The remaining tools were based on international efforts [29,68,119,136,137,139,140].

**Table 2.** Survey tools

Year	Authors	Study Location	Name of Tool	# Items	Validated	Respondents	Description of Tool
2001	Croxson *et al*. [119]	United Kingdom	Unnamed Research and Development Tool	13	No	NA	Individual and organizational level measures focus on research contributions and benefits.
2002	Smith *et al*. [68]	United Kingdom	Wessex Research Network (WReN) Spider	10	Yes	Practice-Based Researchers	Individual level measures focus on value, confidence, supports, activities, and use of research.
2005	Bland *et al*. [87]	United States	Unnamed	150	Yes	Medical School Faculty	Individual, organizational, and leadership level measures focus on characteristics influencing faculty research productivity.
2005	Brocato & Mavis. [20]	United States	Unnamed	43	No	Medical School Faculty	Individual level measures focus on research socialization and environment, researcher characteristics and activities.
2008	Hall *et al*. [112]	United States	Research-Orientation Scale	10	Yes	Investigators	Individual measures focus on the unidisciplinary or cross-disciplinary proclivity of the investigators’ values and attitudes toward research
2012	Holden *et al*. [137]	Australia	Research Capacity and Culture Tool	55	Yes	Allied health professionals	Individual, team and organizational level measures focus on research capacity.
2014	Mills *et al*. [138]	United States	Clinical Research Appraisal Inventory	92	Yes	Clinical researchers	Individual level measures to assess research self-efficacy.
2015	Caminiti *et al*. [29]	Italy	Participation in Research Activities	7	No	Clinicians	Individual level measures to assess research-related training and activities.
2016	The Global Health Network [139]	United Kingdom	TDR Global Competency Framework	13	Yes	Clinical researchers	Individual level measures assess scientific thinking, ethics, quality and risk management, study/site management, research operations.
2017	Brennan *et al*. [136]	Australia	SEER - Seeking, Engaging with and Evaluating Research	46	Yes	Policymakers	Individual level measures assess value, confidence, supports, activities, and research use.
2017	Paiva *et al*. [140]	Brazil	Researchers’ Profile Survey	26	No	Journal authors	Individual level measures assess personal characteristics, research barriers and activities, work-life balance and career satisfaction.

## Discussion

Our review of the literature revealed a range of individual-level measures designed to assess one or more aspects of a researcher’s experience, activities, and outcomes. However, the existing approaches fall short in capturing metrics across all six research categories included in our review. They lack comprehensiveness, with most measures concentrating on a single area. We found limited integration across multiple research categories that collectively represent the depth and breadth of a researcher’s experience and efforts as well as their supports and impact. By developing measures that span our research categories and focus areas, we can capture a more complete picture of a researcher’s activities, personal experiences, and outcomes, rather than focusing narrowly on traditional metrics.

A key observation of our review is the reliance on traditional research productivity metrics such as publications, research grants, and bibliometric indicators. While these are commonly used, they may not fully encompass the complexities of a researcher’s career trajectory or success. Publications, often measured by total counts and citations, may be foundational to many assessments of academic achievement, but they fail to account for the quality of the research, individual contributions, engagement of the community, or the broader influences or impact of the work. In addition, reliance on bibliometric data may result in valuing quantity over quality. As efforts to move away from narrowly measuring research productivity, more holistic approaches to understand the experiences of researchers, the merits of their findings, and their impact may offer a more accurate reflection of a researcher’s efforts and contributions throughout their research career.

Measures related to research funding were common and they provide varied approaches with little consistency. The literature largely focused on grant numbers, dollar amounts, and the nature of awards. There were a few studies that considered the broader context of funding such as the role of the researcher in the grant process. Understanding how financial support such as the type and timing of funding influences a researcher’s ability to attain research independence may be important to measure over time.

This review also revealed a range of output measures designed to capture the variety of activities and skills researchers engage in. These measures often focused on common tasks such as grant writing, presenting research, mentoring students, teaching, and engaging in routine service activities such as peer review. A clear theme across the output measures was the absence of a unified approach to track these diverse efforts, making it more difficult to assess an individual’s progress toward advancing their research career or their broader contributions to their field.

Metrics related to facilitating factors and research barriers were well documented in the literature, particularly as they related to new and emerging researchers. However, despite the varied measures, there is no uniform approach for capturing the supports needed to foster research and mitigate barriers that hinder career progression.

In terms of research mentorship and collaboration, the literature suggests both areas are essential for research success, suggesting a need to include the areas in future effort focused on individual-level researchers. The measures we reviewed related to mentorship tend to focus on the quality of the mentor-mentee relationship, the involvement in meaningful research projects, and the support received, including the effectiveness of the mentor. Similarly, the measures of collaboration we identified tend to focus on the research activities, outputs, contributions of the partners, and the composition of the research team.

Measures related to research impacts continue to evolve and there are a number of useful frameworks that exist. Approaches for assessing ROI have also been widely applied. Yet, more work is needed to create methods to evaluate the individual-level impact of research throughout one’s career trajectory versus the impact of specific research projects. Ongoing efforts to assess a researcher’s outcomes or impact may benefits from the use of additional resources such as the Overton Index policy database (https://www.overton.io/overton-index), a subscription-based resource that provides a robust repository of policy and gray literature, linking individual publications to policy-relevant documents to highlight the impact and translational effects of research [141]. Although this is a subscription-based database that was outside the budgeted resources available for this review, it may be useful to explore for ongoing evaluation efforts of clinical and translational research.

Our review of survey tools also revealed the lack of comprehensive and standardized approaches for assessing individual-level research experience, activities, and outcomes. There are few validated instruments with consistent and standardized methodologies, and many existing tools focus on narrow aspects of a researcher’s career, such as self-reported skills. This complicates the field’s ability to adopt standardized measurement practices.

More comprehensive measurement tools could provide important insights on how funding, career support, and other resources help fortify both the new investigator and the bench-to-bedside/bedside-to-practice pathways in their communities. Future measurement efforts should focus on developing and psychometrically testing new tools that capture the full range of factors that influence a researcher’s experience, activities, and outcomes. Broader methodological approaches could potentially be used to predict research career progression or research success. Future efforts using structural equation modeling or other advanced analytic techniques may help to determine which areas contribute to the experiences of researchers and the outcomes of their research careers.

## Limitations

This review contributes to the evaluation of clinical and translational research by highlighting current approaches and measures used to assess individual-level efforts. Although an attempt was made to widely scan the literature, a systematic review with expanded search terms that focus on “evaluation” and “measurement” may yield additional published measures and tools that were not captured in our narrative review. Our inclusion criteria focused on summarizing published survey tools that were readily available with complete descriptions including their corresponding item wording and response options, thus limiting our scope. We identified several additional tools (e.g., post-program surveys, evaluation surveys) that were excluded because they did not meet these criteria. Finally, we acknowledge previous efforts of CTSAs to document and study a range of bibliometric approaches, including their relationship to programmatic outcomes and collaborative impact. For example, Lellewellyn and colleagues [142] explored CTSA grant-cited publications based on several bibliometrics such as the CNCI along with the JIF, Relative Citation Ratios, and Approximate Potential to Translate. Yu and colleagues [92] outlined a bibliometric approach to evaluating CTSA research outcomes and collaborative impact. We attempted to documentthe range of bibliometric measures available, versus the ways in which they have been used to contribute to the evaluation science of clinical and translational research.

## Conclusion

Assessing changes related to the health research workforce is a priority, yet there are no comprehensive tools that currently exist to measure individual-level attributes, supports, or outcomes among researchers. A range of measures and approaches exist, yet more efforts are needed to create a comprehensive measurement tool that can be widely applicable. Such a tool could help standardize the ways in which a researcher’s experience and institutional supports are studied over time to determine the best avenues for supporting new and early-stage investigators.
